# Conformational Consequences for Compatible Osmolytes on Thermal Denaturation

**DOI:** 10.3390/life11121394

**Published:** 2021-12-13

**Authors:** Nimesh Shukla, Brianna Bembenek, Erika A. Taylor, Christina M. Othon

**Affiliations:** 1 Department of Physics, Wesleyan University, Middletown, CT 06459, USA; nshukla@wesleyan.edu; 2 Department of Physics, Ripon College, Oxford OX44 9EX, UK; bembenekb@ripon.edu; 3 Department of Chemistry, Wesleyan University, Middletown, CT 06459, USA; eataylor@wesleyan.edu

**Keywords:** hydration dynamics, compatible osmolyte, time-dependent Stokes shift, thermal denaturation, disaccharide

## Abstract

Compatible osmolytes are a broad class of small organic molecules employed by living systems to combat environmental stress by enhancing the native protein structure. The molecular features that make for a superior biopreservation remain elusive. Through the use of time-resolved and steady-state spectroscopic techniques, in combination with molecular simulation, insight into what makes one molecule a more effective compatible osmolyte can be gained. Disaccharides differing only in their glycosidic bonds can exhibit different degrees of stabilization against thermal denaturation. The degree to which each sugar is preferentially excluded may explain these differences. The present work examines the biopreservation and hydration of trehalose, maltose, and gentiobiose.

## 1. Introduction

Osmolytes are a class of molecules used by living cells to maintain, regulate, and protect the formation of protein assemblies; they do so indirectly through the modification of the water structure and its activity within the cell [1,2,3,4]. Osmolytes such as disaccharide sugars are hypothesized to alter the structure of water near the surface of proteins, enhancing the stability of the protein structure, and reducing the tendency of the protein to denature under high-stress conditions (elevated temperature, pressure, high salinity, dehydration, and cryogenic temperatures). Osmolytes are a broad class of materials that include sugars, salts, free amino acids, and polyols. These molecules can be present at extreme concentrations (up to several molar) in plant and animals exposed to high-stress conditions and enable the species to endure extreme hydrostatic changes and temperature variation. We began our work by studying disaccharide osmoprotectants in particular, because they appear to be unique in protecting the protein structure from very diverse physical stresses [5,6,7,8,9,10].

Trehalose is the most widely effective polysaccharide osmoprotectant known and is used by many animals to combat stress. Trehalose is a non-reducing disaccharide of glucose linked through an α,α-(1,1)-glycosidic bond. Intriguingly, trehalose is far more effective than similarly sized and chemically analogous disaccharides like sucrose (table sugar) and maltose (malt sugar) [11,12,13], though the source of its enhanced biopreservation properties are widely disputed. Our most recent publications tested some of these water-structuring hypotheses by comparing molecules with a similar size and chemical structure, but differing either in stereochemistry or in the number of water bonds per molecule. We found that, while it appears that for disaccharide molecules the thermal biopreservation capability of the molecules scaled with the water-structuring ability [14], the same was not found for smaller cyclic polyols [15]. 

Most recently we explored smaller compatible osmolytes with a similar size but different hydration number, including glucose, Myo-inositol, and Methyl-α-D-glucopyranoside (Me-Glucose). Glucose is the most abundant food source and commonly employed osmolyte found in nature. Myo-inositol is a small sugar alcohol that is commonly found in drought-stressed plants [16] and is an important renal osmolyte in humans [17,18]. Finally, Me-glucose is an unnatural sugar derivative that was chosen to test the hypothesis that the number of water bonds is directly proportional to efficacy as a biopreservative, as was observed previously for sugars [19]. Using these model systems, we demonstrate that the thermal enhancement of protein structure does not correlate strongly with either the slowing of motion of water molecules in solution or with the number of water bonds the molecule makes with the solvent. However, it may be tightly correlated to the remarkable change in the density of water. Measurements of the partial molar volumes suggest that inositol has an unusually high density of interfacial water as compared to glucose [18]. These measurements suggest that hydration does not scale with the number of hydrophilic groups but scales strongly upon the conformational properties of the system. 

Computational works on trehalose indicate that the relatively rigid α,α-(1→1)-glycosidic bond of trehalose could be a key feature that distinguishes its superior water-structuring capability compared to its stereoisomers and other dimers of glucose [20,21]. In general, the slowdown of hydration dynamics seems to be affected by several factors such as hydration number around the glycosidic oxygen, average number of long-lived hydrogen bonds, total number of long-lived water bridges that results in a lower water activity, intermolecular energy within the first solvation shell, and the number of water molecules within the first solvation shell. The detailed investigation between water density and structure, molecular flexibility, and hydration residence times is relatively unexplored. Experimental measures of hydration are beginning to highlight an unexpected diversity in hydration dynamics (entropic effects) and the strength of the hydrogen bond network (enthalpic effects) in the stabilization of biological molecules.

For this study, we extended our investigations of disaccharide sugars by comparing the hydration dynamics and thermal preservation properties of solutions containing trehalose, maltose, and gentiobiose, which have interesting differences in the hydration. Trehalose has been widely studied and its impacts on water dynamics and structure are well characterized [15,22,23,24,25]. Maltose has similar water density and similar hydration of the glycosidic bond but is also a far less rigid molecule than trehalose [21,26,27]. Gentiobiose has the highest density of water in the solvation layer and the highest hydration number of the glycosidic bond of all of the disaccharides of glucose [21,28]. Using ultrafast fluorescence spectroscopy, we measured of the effects of osmolyte concentration on hydration dynamics. To investigate differences in biological structural preservation, we measured the thermal denaturation for two model protein systems as a function of osmolyte concentration. Lastly, we used molecular simulation tools to isolate possible differences in the co-solute and protein interactions. We find that trehalose exhibited the strongest protection against thermal denaturation. Maltose was similar in its ability to protect against thermal stress, as has been previously demonstrated [29]; however, surprisingly, gentiobiose did not enhance the thermal stability of either protein system. In fact, gentiobiose appeared to weaken the native structure of the proteins and lead to a lowering of the melting point of the proteins investigated. While this was surprising, it is not unprecedented, because a variety of osmolytes have been shown to specifically destabilize proteins under high concentration or high pH [30]. Molecular simulations were performed and demonstrated that, while effective natural osmolytes such as trehalose and sucrose only interact with proteins in a limited fashion, gentiobiose appears to explore the surface of proteins more broadly and creates a larger coating of sugar at the protein interface, consistent with its increased flexibility as compared to trehalose.

## 2. Materials and Methods

### 2.1. Sample Preparation and Characterization

**Figure 1:**
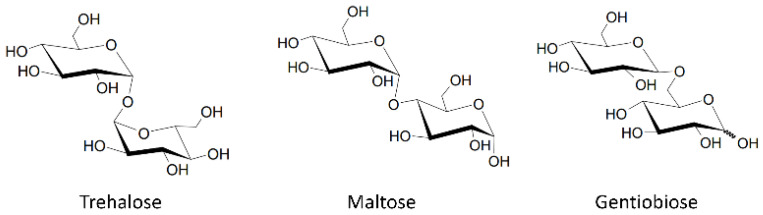
Chemical structure of the three disaccharides investigated in this study.

The chemical structures of the molecules used in this study are given in Figure 1. Trehalose (>99%), maltose monohydrate (>99%), and β-gentiobiose (>85%) were purchased from Sigma-Aldrich, lucifer yellow ethylenediamine (94%) was purchased from Setareh Biotech, and all were used without any further purification. Solutions for fluorescence experiments were prepared from ultrapure 18 MΩ water at concentrations of 200 μM for lucifer yellow ethylenediamine (LYen). All the measurements were taken at room temperature (~21 °C). Staphylococcal nuclease (SNase) was expressed and purified in our lab with the method described by Shortle and Meeker [31] and modifications mentioned by Byrne et al. [32] The protein concentration was determined to be 10.2 mg/mL using the extinction coefficient ε280 = 0.93 mg/mL·cm^2^ [33]. The protein samples were stored at −80 °C in water. Prior to performing melting experiment, the samples were thawed and adjusted to final concentration of 1mg/mL. Hen egg white lysozyme (>90%) was purchased from Sigma-Aldrich, and samples for circular dichroism were prepared according to the protocol described by Greenfield et al. [34]. Samples were prepared in a 10 mM phosphate buffer, pH 7.2, and stored at 4 °C for up to one week prior to use. The final concentration of lysozyme samples was 0.83 mg/mL.

### 2.2. Steady State Spectroscopy

Circular dichroism (CD) spectra from 200 nm to 250 nm were measured to investigate the secondary structure of proteins. CD melting experiments were performed on a Jasco spectropolarimeter (model J-810, Jasco International, Tokyo, Japan). The temperature was varied from 30 °C to 70 °C in 2 °C increments for SNase in a quartz cuvette of path length 0.2 cm. The temperature was varied from 50 °C to 94 °C in 2 °C increments for lysozyme in a quartz cuvette of path length 0.01 cm. The measurements were done in triplicate using a reference buffer containing an identical concentration of sugar. The buffer CD spectrum was then subtracted from the protein spectrum to obtain a true CD spectrum of proteins at each temperature and co-solute concentration. The spectra were fit using the Dichroweb [35] and Bestsel programs [36]. The melting temperature of the system was taken by examining the ellipticity of the spectrum at 222 nm, which corresponds to the absorption minimum due to alpha helical secondary structure of the protein. The ellipticity at 222 nm was then plotted as a function of temperature and fit using sigmoidal curve to obtain the melting point of the protein. The melting temperature is defined as the point of inflection in the fit of sigmoidal curve. This absorption feature has been shown to accurately reflect the melting temperature of a wide variety of protein systems as compared to other experimental techniques [34,37].

Lucifer yellow ethylenediamine was chosen due to its solubility in water, sizeable dynamic Stokes shift, and because its excitation and emission both occur in the visible regime. These properties make it easy to work with for the measurement of hydration dynamics. Absorption spectra were recorded on a spectrophotometer (Beckman-650, Beckman Instruments, Fullerton, CA, USA). The emission spectra were recorded on a spectrometer (Ocean Optics-QE65000, Ocean Optics Inc., Dunedin, FL, USA). The absorption/emission maximum of LYen in pure water was measured as 426.1 ± 0.5 nm and 540.9 ± 0.5 nm spectra, respectively. The absorption/emission maxima for concentrations of 0.5 M for all three sugar co-solutes were measured to be the same, within instrumental error.

### 2.3. Fluorescence Spectra Measurements

Lifetime measurements conducted on a time-correlated single photon-counting apparatus described in detail elsewhere [38]. The lifetime of LYen in pure water was measured as 5.9 ± 0.1 ns. The lifetime of LYen was recorded for sugar concentrations of 0.25 M. For all three disaccharides, the lifetime was measured as 6.0 ± 0.1 ns. The lifetime was measured independently and incorporated into the global fit of hydration dynamics.

The measurement of hydration dynamics for LYen is described in detail in Shukla et al. [38]. Briefly, a high-energy oscillator (Coherent Chameleon Ultra II, Coherent Inc., Santa Clara, CA, USA) was used as the source of the fundamental beam. The laser output was tuned to 850 nm, with ~140 fs pulses at 80 MHz, giving 30 nJ/pulse. The output beam was split into equal parts to generate the pump and gate pulse. The excitation beam was frequency doubled using a 1.0 mm thick LBO crystal to provide 425 nm pump pulses attenuated to 1.2 nJ. The pump pulses were focused onto a rotating sample cell (1 mm thickness). The up-converted signal was focused on the entrance slit of a 0.5 mm monochromator (Oriel Instrument-77250 series, Newport Corporation, Irvine, CA, USA) equipped with a photomultiplier tube (Hamamatsu H9305-04, Hamamatsu Photonics, Iwata City, Japan) at the exit slit. The photomultiplier output was amplified using a lock-in amplifier (SR530). The up-converted transients were collected for 10 different wavelengths across the emission spectra. Sample fluorescence intensity and spectra were monitored using a fiber-coupled spectrometer (Ocean optics-QE65000, Ocean Optics Inc., Dunedin, FL, USA) before and after the collection of up-conversion transients to check for sample degradation and photobleaching. No difference between the spectra was observed. The fluorescence transients were globally fit using a sum of three exponential functions convoluted with the Gaussian instrumental response function (IRF) [39,40].

### 2.4. Autodock Molecular Simulations

The ligand docking simulations are a method to predict the preferred conformation or an orientation of ligand molecule with a macromolecule upon binding using a minimization of energy of the ligand/macromolecule complex. We chose reference enzyme systems where a bound substrate was also reported. This was done to provide a reference binding energy for a known substrate to calibrate the strength of the interactions of the co-solute and to verify the proper simulation and docking of the substrate at the active site. The proteins used in this study along with their PDB code were hen egg-white lysozyme (1HEW) [41], Subtilisin DY (1BH6, a random mutant of subtilisin Carlsberg) [42], wild-type Staphylococcal nuclease (4WOR) [43], and Thrombin Activatable Fibrinolysis Inhibitor (5LYD) [44]. For convenience, from now onwards, we will refer to these proteins as lysozyme, Subtilisin, SNase, and Thrombin, respectively. Docking simulations were performed using a publicly available ligand docking software Autodock Vina [45] along with AutoDockTools. The structure of substrates of all proteins were downloaded from the RCSB Protein Data Bank, and the structures of the disaccharides were prepared using ChemDraw (Perkin Elmer Informatics, Waltham, MA, USA). Ligands conformations were energy minimized using MOPAC [46] before seeding the conformation in Autodock Vina. All the rotatable bonds were kept mobile. The metal ions reported in crystal structure of proteins were kept present for all docking simulations. Proteins structures were kept rigid, and the value of exhaustiveness parameter was fixed to 100. A total of 400 poses were generated for each ligand by running Autodock Vina 20 times, each time using a random seed conformation and a random iteration parameter. To ensure the validity of docking algorithm used by Autodock Vina, substrates were removed from crystal structure of proteins, seeded, and then docked to their respective proteins. All simulations of the disaccharides were performed after making sure that substrate of each protein was docking correctly at its active site as reported in the crystal structure. For highlighting the local hydrophobicity on the protein surface, a YRB color code scheme proposed by Hagemans et al. [47] was produced using the python script provided in their work. The visualization, editing, and printing of docking results were done using PyMol [48].

## 3. Results

### 3.1. Melting Temperature of Proteins in Presence of Disaccharide Co-Solutes

We conducted experimental measurements of the structural enhancement of proteins in the presence of trehalose, maltose, and gentiobiose for two different protein models. Lysozyme is a small, 129-residue enzyme that has been widely studied as a model for protein folding and aggregation [49]. Lysozyme*’*s secondary structure comprises four alpha (and one 3–10) helices and five beta strands. The enzyme functions by hydrolyzing glycosidic bonds in peptidoglycans. The structure of lysozyme is well understood and it is frequently employed in molecular simulations and has been studied as a model for protein–trehalose interactions [19,27,50]. We chose lysozyme as a model system, due to its commercial availability at high purity, and because of its characterization as a model system. Lysozyme can be thermally denatured at a melting temperature of 72 °C [51,52] at a neutral pH. However, lysozyme can bind to the glycosidic bond and may interact with our disaccharide co-solutes. Staphylococcal nuclease (SNase), is a small enzyme of 149 amino acids that cleaves either DNA or RNA substrates. SNase has three long alpha helices and a 5-stranded, barrel-shaped beta sheet, and is widely employed for protein folding experiments due to its two-state unfolding process and comparably low melting temperature of 53 °C [32].

We previously published data for the enhancement of thermal stability in the presence of the natural compatible solutes for trehalose, sucrose, glucose, and myo-inositol for these protein model systems [14,15]. Trehalose has repeatedly shown a strong enhancement of thermal biopreservation giving a concentration-dependent increase in melting temperature of 10 °C/Mol for SNase and 7 °C for lysozyme. This enhancement is higher than that demonstrated in sucrose, and is matched only by that of myo-inositol, which is widely employed in the plant kingdom as a mechanism for drought tolerance [16]. We use trehalose as a benchmark to compare the thermal preservation abilities of two other disaccharides of glucose: maltose and gentiobiose. Maltose has been demonstrated to be an effective compatible osmolyte against thermal denaturation [29]. Both maltose and gentiobiose are known to be non-accumulating osmolytes [53] that are catabolized during early exponential growth, contributing indirectly to enhance the levels of two endogenously synthesized osmolytes, glutamate and N-acetylglutaminylglutamine amide, in bacterial species. However, there is no indication in the literature that gentiobiose itself is capable of protecting proteins from thermal denaturation. Here, we provide direct evidence that it is not an effective compatible osmolytes using two model protein systems. 

**Figure 2:**
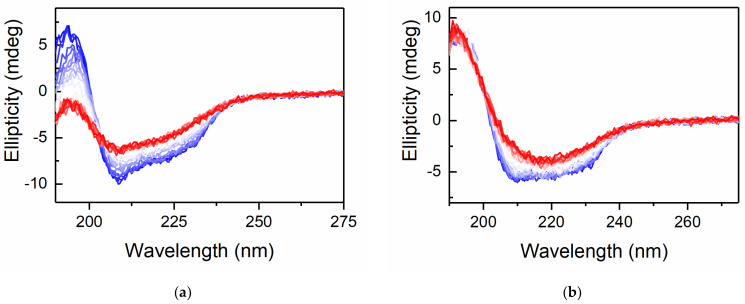
(**a**) Circular dichroism spectra for lysozyme in a 10 mM phosphate buffer, pH 7.2 and (**b**) in the same buffer with the addition of 0.2 M gentiobiose. The temperature ranges from 50 °C (blue lines) to 94 °C (red lines) in two-degree increments.

Figure 2 shows the thermal denaturation of lysozyme at a concentration of 0.83 mg/mL in a phosphate buffer and in the same buffer with 0.2 M gentiobiose co-solute concentration. We note that the initial structure at 50 °C is different for these two samples. In buffer lysozyme exhibits as substantial absorption feature at 208 nm, which is typical for lysozyme at this concentration and temperature. This is dramatically reduced in the presence of gentiobiose, as is the overall ellipticity of the structure. Furthermore, while lysozyme in buffer exhibits an overall reduction in structural features for both helical and β-sheet with increased temperature, in the presence of gentiobiose, the structure transitions to a different structural motif as the temperature increases. These spectra appear more rounded and have a minimum close to 218 nm, which is consistent with an anti-parallel β-sheet [34]. In fact, this structural transition from α-helical to β-sheet has been observed for lysozyme in the presence of 90% ethanol [54]. This transition is attributed to amyloid formation in the protein solutions. We therefore could not attribute an accurate melting temperature trend to solutions containing gentiobiose, as they are transitioning from one folded state to a new intermediate, non-native pathway in the presence of the co-solute gentiobiose. This behavior has never been observed for trehalose samples, including those that were stored for a significant period of time. At the highest concentrations of maltose, we did see a slight diminishment of helical content near low temperatures, and could not rule out the possibility of amyloid formation for these samples; however, the effect is much weaker than that of gentiobiose.

**Figure 3:**
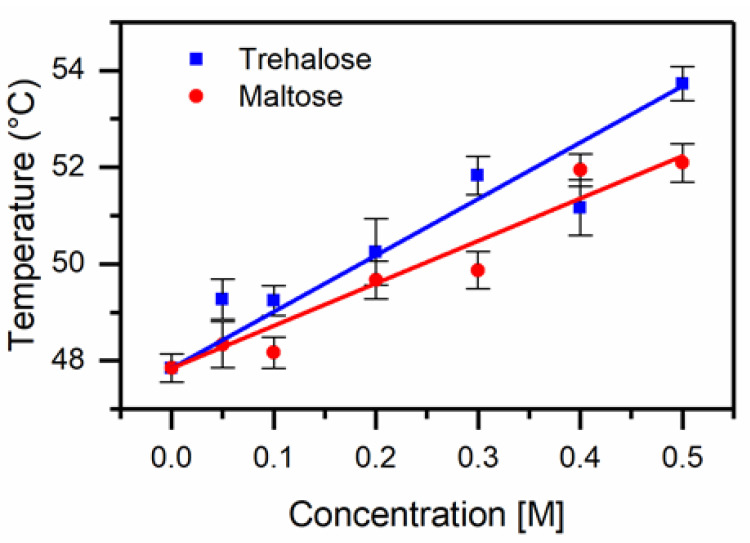
Melting temperature of SNase as a function of concentration for trehalose and maltose. We found a slope of 11.0 +/−0.7 °C/M for trehalose and 8.8 +/−0.6 °C/M for maltose.

**Figure 4:**
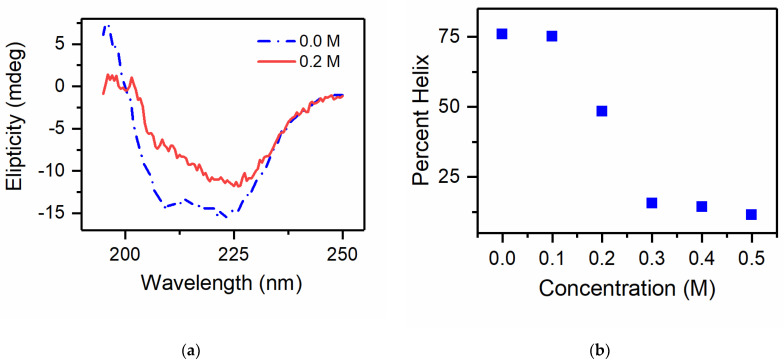
(**a**) Circular dichroism spectra for SNase in a water (blue line) and in a solution of 0.2 M gentiobiose in water (red line). (**b**) The percent helicity as calculated using BestSel protein structure fitting algorithm as a function of gentiobiose co-solute concentration (right panel).

To further investigate the interaction of these co-solutes, we measured the thermal denaturation for an additional protein model SNase. Both trehalose and maltose increased the thermal stability of SNase substantially. We found that trehalose was more effective at protecting the helical structure of SNase, see Figure 3; however, both were effective compatible osmolytes for this protein system. Gentiobiose did not enhance the thermal stability of SNase; in fact, the melting temperature of the system was lowered for low concentrations of gentiobiose, and a significant reduction of helical content was observed for SNase at room temperature for concentrations higher than 0.2 M, see Figure 4. It appears that above a critical concentration of gentiobiose, the protein structure unfolds, exhibiting a significant drop in helical content. Both protein systems investigated exhibited a destabilizing interaction between gentiobiose and the native protein structure at room temperature, which was not present for either maltose or trehalose.

### 3.2. Hydration Dynamics of Disaccharide Solutions

**Figure 5:**
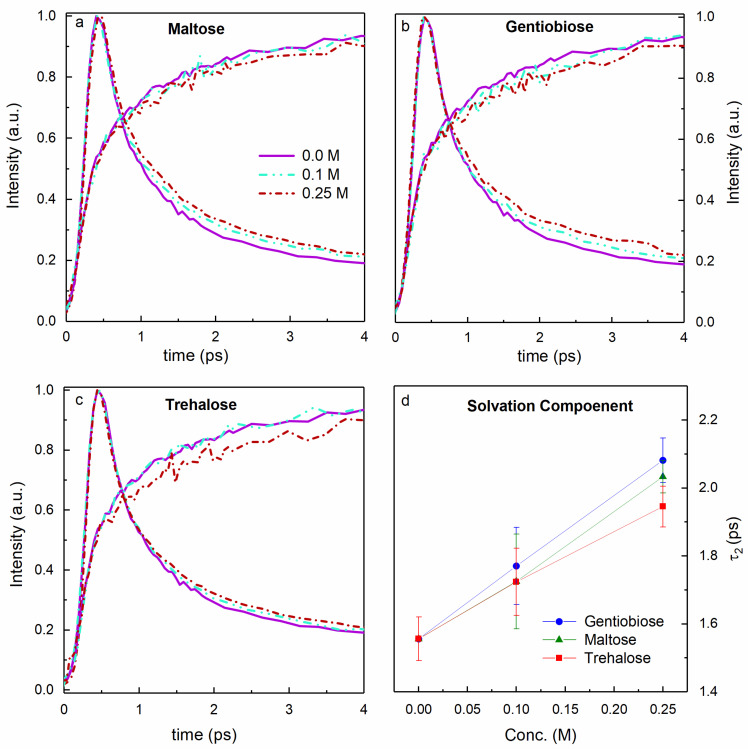
Example up-conversion transients at 460 nm (fast decaying curves) and at 640 nm (slow rising curves) for (**a**) maltose, (**b**) gentiobiose, and (**c**) trehalose. (**d**) Increase in the solvation time constant of LYen with respect to the concentration.

Time-resolved up-conversion spectroscopy was used to measure changes in the dynamic behavior of water in co-solute solutions up to 0.25 M. Time-resolved fluorescence experiments have proven extremely sensitive to the hydration dynamics in co-solute solutions. Though they are not probe free as other hydration experiments [19,24,55,56,57], and they are extremely valuable at detecting small changes in hydration and at low solute concentration. Therefore, when investigating solutes that are as chemically similar as the solutes presented in this study, fluorescence up-conversion represents an ideal tool for detecting hydration changes. Raw up-conversion transients for maltose, gentiobiose, and trehalose are shown in Figure 5. The time constants obtained by performing the global fitting analysis are summarized in Table 1.

We found that the hydration dynamics for these three disaccharides is virtually indistinguishable in the low concentration range, see Table 1. The fast timescale, τ_1_, is generally attributed to fast vibrational and librational motions of the solvent and is independent of co-solute concentration or moiety. The timescale on the order of a picosecond, τ_2_, is attributed to translational and rotational relaxation of the solvent. The measurements lie within uncertainty of each other using this measurement technique. The steady-state absorption, emission, and fluorescence lifetime measurement were also identical, and indistinguishable from the lifetime measured in pure water as shown in the Appendix A. The lack of any change in the lifetime of LYen indicates no direct interaction between the probe and the co-solute molecules. The measurement of hydration relaxation indicates that the interactions between the co-solute and water are very similar in these solutions, and that there is no meaningful difference in the bulk hydration dynamics for these solutes. This is not surprising given the chemical similarity of these solutes. We expect similar hydrodynamic volume, and all disaccharides engage in a similar hydrogen-bonding capacity. This implies the reduction in biopreservation properties of gentiobiose as compared to maltose and trehalose is not due to a change in its ability to alter the water structure or dynamics. 

### 3.3. Molecular Simulation of Protein–Co-Solute Interactions

**Figure A4:**
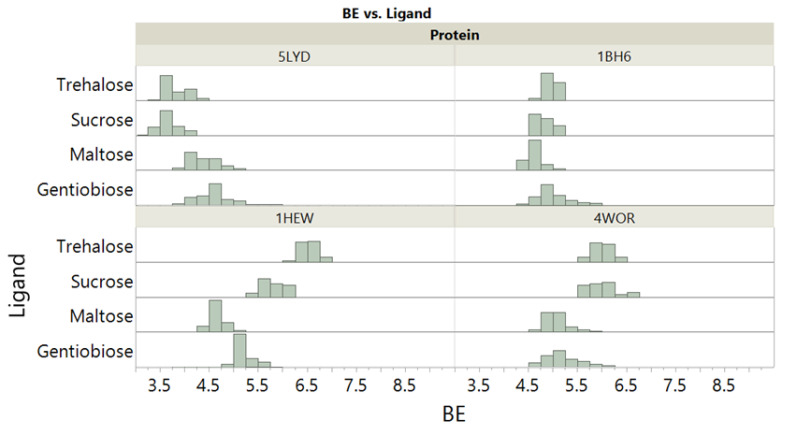
Mean Binding Energies for the co-solutes and proteins presented in Figure A2 and Figure A3.

To shed light on the difference between the interaction of these sugars with proteins, ligand docking simulations were performed on four model enzyme systems. At first, all proteins were docked using their substrate as ligand. This provided a reference energetic binding level to compare to the co-solute interactions. The proper binding of the ligand to the binding site reported in the crystal structure also provides a good test to ensure the simulation is operating as expected. In order to observe a range of events, we generated 400 docking poses for each substrate, and the mean and least binding energies are reported in Figure A4. We found that the orientation of least energy conformation of each substrate matched very well with the orientation of substrate reported in the crystal structure. We also observed that majority of substrate conformations actually ended up binding at the active site [58].

**Figure 6:**
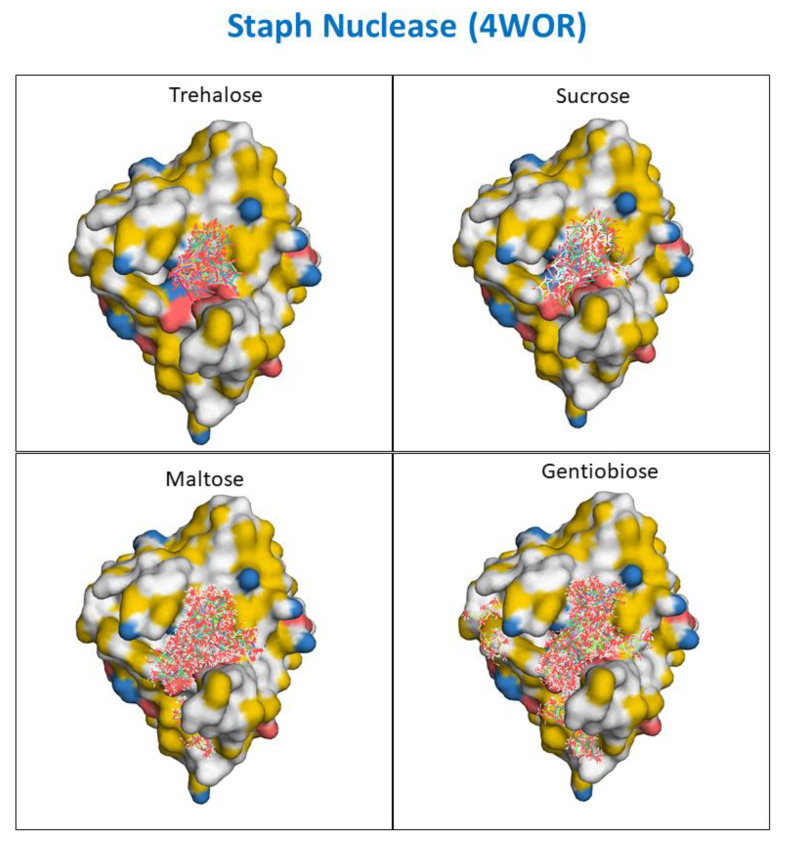
Autodock Vina simulations of SNase interactions with the disaccharide molecules trehalose, sucrose, maltose, and gentiobiose.

We docked the disaccharides to each of the proteins systems. We observed that trehalose, maltose, and gentiobiose all bound to the proteins with a lower calculated affinity than did their natural substrates. This was expected, as these enzymes have evolved to be highly selective with their target substrates. We also observed that the co-solutes that nature selected as highly effective compatible osmolytes, namely trehalose and sucrose, only appeared to interact with the model enzyme systems near the active site (see Figure 6 and the figures within the Appendix A). This is not surprising, as in our simulations, interaction is modeled as a global optimization to a rigid protein structure, which limits the number of possible interaction sites and the size of our simulation. Furthermore, in the program, the ligand is forced to bind somewhere by the end of the simulation, regardless of whether this would happen in solution or not. In all likelihood, the sugar would be out-competed or would prefer to interact with water than bind to the active site of the enzyme in solution. 

Both maltose and gentiobiose appeared to explore the surface of the model protein systems more extensively and interacted in a larger number of sites than the natural osmolytes trehalose and sucrose. These interactions were weak. However, there appeared to be a greater number on non-specific interactions between gentiobiose and all four of the proteins investigated. This behavior appears consistent with a change in the nature and degree to which gentiobiose is preferentially excluded from the protein surface. 

## 4. Discussion

The thermal denaturation data in conjunction with the molecular simulation data imply that gentiobiose is no longer preferentially excluded from the protein interface. In fact, due to the similarity in concentration-dependent behavior with ethanol in a lysozyme system, the behavior of gentiobiose appears to be creating a weakly bound coating of co-solute around the surface of the protein. Since we observed that the protein destabilization of gentiobiose increases with increasing disaccharide concentrations, we hypothesize that this results in a weakening of the hydrophobic effect, which stabilizes the native structure in solution. This enables the protein to enter the intermediate amyloid state in the case of lysozyme, and partially denatures the protein in the case of SNase. To our knowledge, this is the first time anyone has explored the interaction of gentiobiose with protein structures. We initially assumed, due to the chemical homology with trehalose and maltose, that gentiobiose would behave as a moderate-to-weak osmolyte. The detrimental concentration-dependent behavior of gentiobiose was a complete surprise; however, since it is not the first case of a polyol leading to protein destabilization, further work is needed to determine if this is a common phenomenon amongst disaccharides. 

This work emphasizes that the most essential behavior for a successful compatible osmolyte is its ability to remain highly solvated. Trehalose has the highest energy of solvation and longest water residence time of all of the disaccharides of glucose [21]. Gentiobiose by comparison has a much weaker interaction with water. It is interesting that these differences do not appear more substantially in the time-resolved hydration dynamics. We observed negligible differences in the dynamics of water in these co-solute solutions. This implies that the hydration dynamics in bulk are dominated by hydrodynamic volume [59] and the hydrophilic nature of the solute, [15,58] but are not highly dependent on interfacial energy or dynamics near the surface of the solute. 

## Figures and Tables

**Table 1:** Data for hydration dynamics in the presence of co-solutes as measured by time-resolved 286 fluorescence spectroscopy.

Disaccharide	Concentration [M]	Lifetime (ns)	τ_1_ (ps)	τ_2_ (ps)
Maltose	0.10		300 ± 40	1.73 ± 0.14
	0.25	6.2	310 ± 10	2.04 ± 0.05
Gentiobiose	0.10		290 ± 30	1.78 ± 0.12
	0.25	6.1	310 ± 20	2.09 ± 0.07
Trehalose	0.10		280 ± 30	1.73 ± 0.10
	0.25	6.1	270 ± 20	1.95 ± 0.06

**Figure A1:**
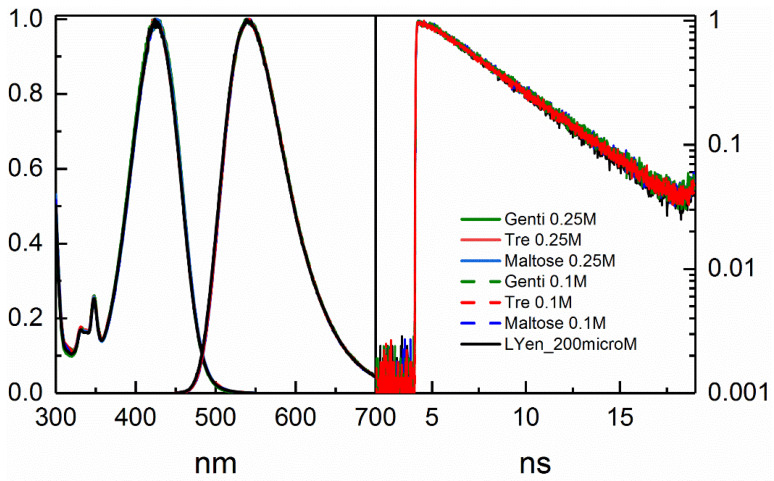
(**left**) Steady-state absorption and fluorescence emission spectra for 200 µM LYen in a solution with pure water (black), 0.25 M trehalose (red), 0.25 M maltose (blue), and 0.25 M gentiobiose (green), exhibiting no shift in spectral absorption or emission in presence of co-solute. (**right**) Fluorescence lifetime transients for 200 µM LYen in solution with pure water, trehalose, maltose, and gentiobiose. The lifetime is indistinguishable in the presence of co-solutes.

**Figure A2:**
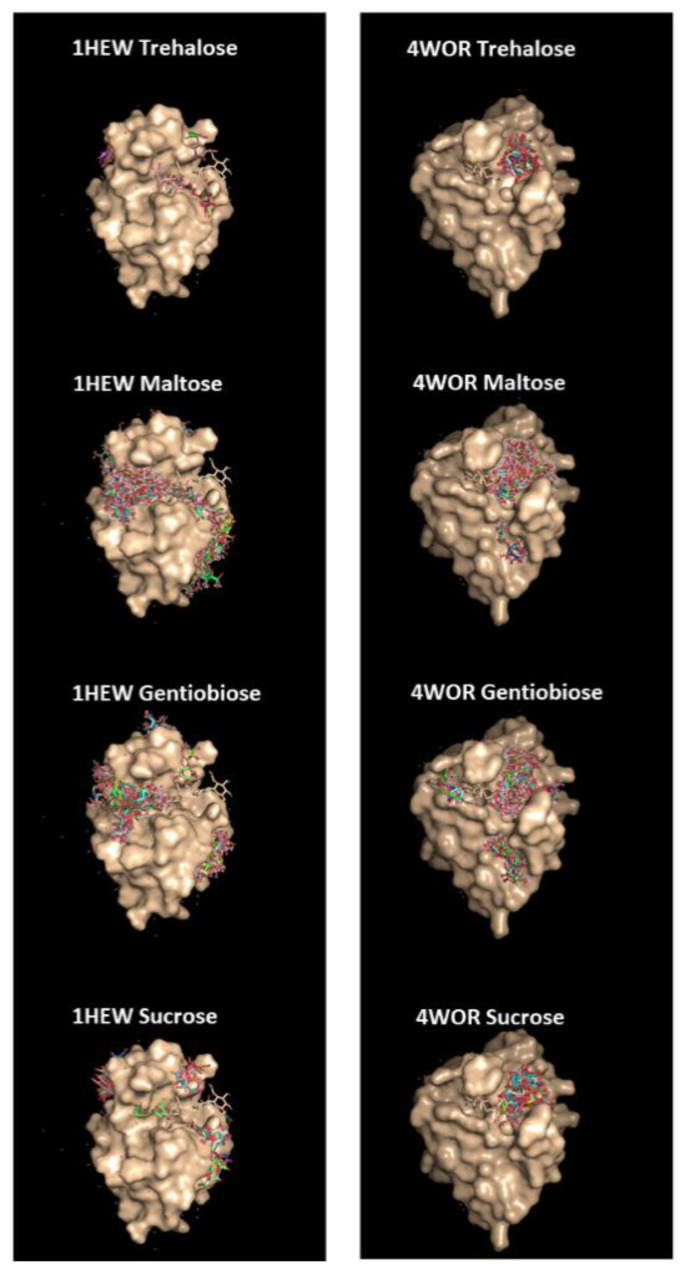
Molecular simulations for interactions of the co-solutes trehalose, maltose, gentiobiose, and sucrose with the model proteins hen egg-white lysozyme (1HEW) and SNase (4WOR). Sugars appear in color, while the protein and bound ligand appear in beige.

**Figure A3:**
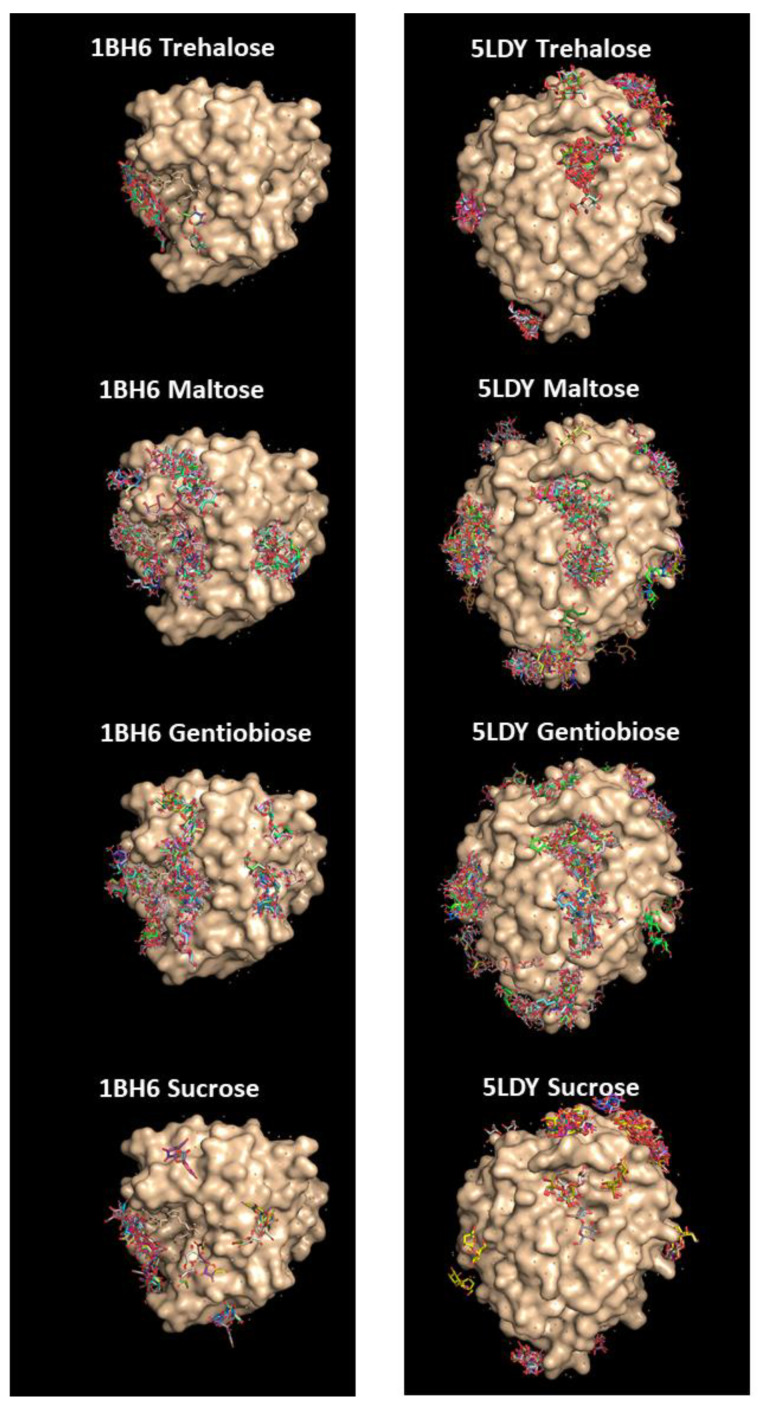
Molecular simulations for interactions of the disaccharides with Subtilisin (1BH6) and Thrombin (5LDY). Sugars appear in color, while the protein and bound ligand appear in beige.

## Data Availability

Not applicable.
